# A new method to estimate branch biomass from terrestrial laser scanning data by bridging tree structure models

**DOI:** 10.1093/aob/mcab037

**Published:** 2021-03-07

**Authors:** Man Hu, Timo P Pitkänen, Francesco Minunno, Xianglin Tian, Aleksi Lehtonen, Annikki Mäkelä

**Affiliations:** 1 Department of Forest Sciences, University of Helsinki, Latokartanonkaari 7, Helsinki, Finland; 2 Institute for Atmospheric and Earth System Research/Forest Sciences, Faculty of Agriculture and Forestry, University of Helsinki, Helsinki, Finland; 3 Natural Resources Institute Finland (Luke), Latokartanonkaari 9, Helsinki, Finland

**Keywords:** Branch biomass, terrestrial laser scanning, tree-structure model, individual branch attributes, quantitative structure model

## Abstract

**Background and Aims:**

Branch biomass and other attributes are important for estimating the carbon budget of forest stands and characterizing crown structure. As destructive measuring is time-consuming and labour-intensive, terrestrial laser scanning (TLS) as a solution has been used to estimate branch biomass quickly and non-destructively. However, branch information extraction from TLS data alone is challenging due to occlusion and other defects, especially for estimating individual branch attributes in coniferous trees.

**Methods:**

This study presents a method, entitled TSM_tls_, to estimate individual branch biomass non-destructively and accurately by combining tree structure models and TLS data. The TSM_tls_ method constructs the stem-taper curve from TLS data, then uses tree structure models to determine the number, basal area and biomass of individual branches at whorl level. We estimated the tree structural model parameters from 122 destructively measured Scots pine (*Pinus sylvestris*) trees and tested the method on six Scots pine trees that were first TLS-scanned and later destructively measured. Additionally, we estimated the branch biomass using other TLS-based approaches for comparison.

**Key Results:**

Tree-level branch biomass estimates derived from TSM_tls_ showed the best agreement with the destructive measurements [coefficient of variation of root mean square error (CV-RMSE) = 9.66 % and concordance correlation coefficient (CCC) = 0.99], outperforming the other TLS-based approaches (CV-RMSE 12.97–57.45 % and CCC 0.43–0.98 ). Whorl-level individual branch attributes estimates produced from TSM_tls_ showed more accurate results than those produced from TLS data directly.

**Conclusions:**

The results showed that the TSM_tls_ method proposed in this study holds promise for extension to more species and larger areas.

## INTRODUCTION

Tree biomass estimates are essential in modelling gross primary production of forest stands and understanding what role forests play in the global carbon cycle. Branch biomass, as an important component of tree above-ground biomass, can help to reflect how climate change influences carbon allocation patterns (Delucia *et al.*, 2000). Individual branches, on the other hand, can reflect the vertical crown biomass distribution, or have implications for timber quality via knots (Helmisaari *et al.*, 2002; Mäkinen and Mäkelä, 2003), which are essential for targeting harvest operations and for optimizing thinning strategies in sustainable forest management.

Traditionally, destructive measurements are required for establishing and modelling tree structure, biomass and growth. As field work is time-consuming and labour-intensive, terrestrial laser scanning (TLS) offers a solution for the measurement of tree structure quickly and non-destructively. Recent studies have reported that TLS can successfully produce various tree structural variables, showing great potential to remedy the disadvantages of destructive measurement in forest research (Gonzalez De Tanago *et al.*, 2017; Stovall *et al.*, 2017; Atkins *et al.*, 2018).

Previous studies have estimated total branch biomass through allometric or theory-based equations, with which variables can be derived from easily measured variables (e.g. diameter at breast height, tree height, crown length) (Marklund, 1988; Mäkelä, 1997; Medhurst *et al.*, 1999; Lehtonen *et al.*, 2004*a*; Repola, 2009), or summing individual branch biomass estimates using empirical models (Lehtonen *et al.*, 2004*b*). Coupling biomass estimate models and TLS data, it is possible to estimate tree biomass with non-destructive measurement (Kankare *et al.*, 2013; Stovall *et al.*, 2017; Momo Takoudjou *et al.*, 2018; Lau *et al.*, 2019). Regarding specific branch biomass estimates, most approaches are either based on allometry models with TLS-derived parameters (Hauglin *et al.*, 2013; Gonzalez De Tanago *et al.*, 2017), or use branch volume derived from tree quantitative structure models (TreeQSMs) multiplied by branch wood density (Lau *et al.*, 2018). These methods consider tree-level branch biomass, while individual branch attributes have been more difficult to estimate. The most important reasons are poor visibility due to branch overlapping, and long distance from the scanner, which increases the footprints of beams and results in more occlusions. Previously, Pyörälä *et al.* (2018*a*) assessed branch structure and evaluated performance using manual point cloud measurements, coming to the conclusion that it remains challenging to capture the full branching structure with TLS alone. Some studies reported that large uncertainty may occur when extracting branches of length <5 cm or diameter <10 cm from point clouds (Kaasalainen *et al.*, 2014; Lau *et al.*, 2018). In addition, most current research on branches has been performed on deciduous trees, and TLS scans were collected during the leaf-off season (Lau *et al.*, 2019; Vicari *et al.*, 2019; Wang *et al.*, 2019; Zimbres *et al.*, 2020). Given that coniferous trees are evergreen and have denser branches that overlap vertically, branch extraction directly from TLS could present greater difficulties and challenges within boreal conditions, which cover 70 % of the coniferous forests in the world (UN-ECE/FAO, 1985).

One possible solution to improve individual branch estimation would be to bridge individual branch prediction models with TLS data. Previously, a few studies have reported practical models for estimating individual branch biomass or other attributes: Mäkinen and Mäkelä (2003) developed statistical models to estimate branch basal areas at different heights within the live crown; Lehtonen *et al.* (2004*b*) proposed a mixed effect model to estimate the dry weight of each branch using branch diameter as the only variable, with unbiased predictions; and Helmisaari *et al.* (2002) used branch and length and relative height of the crown to estimate the individual branch biomass. Additionally, pipe model theory (PMT) premises that stem cross-sectional area at any height of the stem is proportional to the cumulative branch basal area above this height (Shinozaki *et al.*, 1964*a*, *b*; Valentine, 1985). Moreover, Pitkänen *et al.* (2021) have proposed a novel method based on TLS data, which significantly improved estimates of stem taper curve and provided accurate stem diameter at a given height. Bridging these studies with TLS data could enable us to estimate individual branch biomass.

Taking account of the current limitations in estimating individual branch biomass with TLS, our study aims to develop a new method (TSM_tls_) for individual branch biomass estimation that bridges tree structure models and TLS data. The TSM_tls_ method is an improvement on using TLS data alone to estimate branch biomass, where branch information extracted from cloud point data is often inaccurate or even missing because of noisy scan data. We estimated the tree structural model parameters from 122 Scots pine (*Pinus sylvestris*) trees destructively measured earlier in southern Finland. The TSM_tls_ method was tested using six Scots pine trees that were first TLS-scanned and later destructively measured in Lapinjärvi, Finland. The specific objectives of the study were the following:

(1) to develop a new method (TSM_tls_) for estimating individual branch attributes from TLS data;
(2) to evaluate the accuracy of the individual branch biomass derived by TSM_tls_ against the destructive tree measurement; and
(3) to compare the tree-level branch biomass estimate derived by TSM_tls_ with other TLS-based methods against the destructive tree measurement.

## MATERIALS AND METHODS

### Study materials

Two data sets were included in our study, referred to as dataset I and dataset II below. Dataset I included destructive measurements and TLS data collection. The field work was conducted in August 2018 at the Latokartano research forest, Lapinjärvi (60°37′ N, 26°10′ E). Six old Scots pine (*Pinus sylvestris*) trees from three plots were scanned before felling. The trees came from two site types, characterized based on their ground vegetation as mesic heath forest and sub-xeric heath forest (Table 1).

**Table 1.** Detailed information on the sample trees from dataset I

Plot	Tree no.	Site type	Site description	Age	*H* (m)	HC (m)	DBH (cm)	*N*_*b*_	DC (cm)
1	11	MT	Mature thinned site	91	29.62	7.61	24.0	103	15.1
	12			97	30.88	10.92	28.1	96	16.9
2	21	VT	Seedling tree stand with seed trees	108	24.01	11.07	29.6	94	19.3
	22			105	24.88	8.56	31.3	64	18.1
3	31	CT	Mature trees on a cliff	108	23.03	9.56	35.4	89	22.2
	32			113	23.69	12.6	40.5	96	34.0

MT, mesic heath forest; VT, sub-xeric heath forest; CT, xeric heath forest; *H*, tree height. HC, crown length; DBH, diameter at breast height; *N*_*b*_, number of all living branches above the crown base; DC, diameter at crown base.

Dataset II refers to the VAPU database (collected by the Finnish Forest Research Institute) (Korhonen and Maltamo, 1990), which is based on biomass measurements and detailed information about tree dimensions. Destructive tree sampling was established from 94 plots during 1988–90. Lehtonen *et al.* (2004*b*) have described the sampling design in more detail. A total of 122 Scots pine trees and 13 947 branches were included in our analysis. For each sample tree, destructive measurements were performed using a protocol identical with that of dataset I.

### TLS sampling and field data collection

#### TLS data collection and pre-processing steps.

 For dataset I, the TLS scans were collected with a Leica Scan Station P40, which is a time-of-flight LiDAR scanner. In each plot, six scan positions were set up and co-registration was performed with Leica’s Cyclone software (Leica Geosystems, 2016). Once the sample trees were extracted from the point cloud, a TreeQSM algorithm was applied to reconstruct the stem and branch structures. TreeQSM is a model to reconstruct trees as hierarchical collections of cylinders and to describe basic branch structure, geometric and volumetric properties quantitatively. It has been successfully applied to derive tree volume and structure in many studies (Raumonen *et al.*, 2013, 2015; Lau *et al.*, 2019; Raumonen, 2019; Krishna Moorthy *et al.*, 2020). Using TreeQSM, it is possible to compute various geometrical stem and branch properties, such as tree height (H), diameter at breast height (DBH), branch diameter, branch length and branch volume.

#### *Reference measurements from destructive trees*.

For dataset I, breast height was marked, and DBH was measured before felling. For every branch originating from the trunk, we measured the diameter and the distance from the treetop. Stem diameter was measured at eight points along the stem: the stump point, 1.3 m, crown base, half of the crown base and 25, 50, 75 and 90 % of crown length. Crown base was recorded as the height of the lowest branch and a maximum of one dead whorl above was allowed. A total of 811 individual branches (including 542 living branches above the crown base and 269 dead branches) were measured. Sample branches with measured cross-diameters were selected randomly from each tenth of the crown length. A total of 60 sample branches were taken to the laboratory and dried in paper bags at 105 °C for 48 h to determine the dry branch biomass (needles were removed from branches after drying).

### Branch biomass estimation from TLS data

#### Overview of the TSM_tls_ method.

**Fig. 1.**
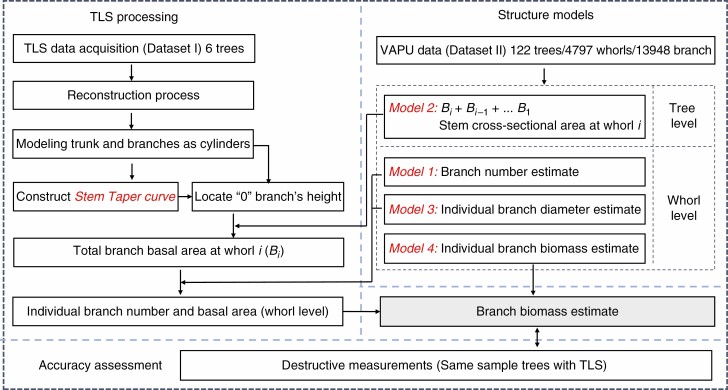
Framework of TSM_tls_ developed in this study to estimate branch biomass from TLS data and tree structure models.

The TSM_tls_ method integrated several models, including TLS-based and PMT-based tree structure models. Using this method, branch biomass was estimated using a five-step process (Fig. 1) in which we:

(1) extracted the height of each individual branch attached to the trunk (‘0’ branch) as observed by TreeQSM (TLS data from dataset I);
(2) clustered the extracted ‘0’ branches to each whorl using a criterion generated from dataset II and estimated branch number at each whorl (Model 1);
(3) estimated the total branch basal area at each whorl using a PMT-based tree structure model (stem taper curve and Model 2);
(4) estimated individual branch basal area at each whorl using tree structure models (Model 3); and
(5) estimated individual branch biomass using a linear mixed model (Model 4).

#### Stem taper curve.

 We estimated the stem taper curve by modelling the stem first as cylinders based on the TreeQSM method (Raumonen *et al.*, 2013, 2015) and the results from Pitk.nen *et al.* (2021). This process included three principle steps:

(1) co-registered TLS data were used to model the stem roughly as a set of connected cylinders, based on their best fit and with no prior expectations with regard to the stem dimensions;
(2) the cylinder model was used to split the stem into thin slices, which were further processed to refine their diameter estimates; and
(3) slice diameters, together with known dimensions (DBH, and H with a diameter of zero), were applied to construct the final spline-based taper curve. This taper curve was then used to calculate the stem diameter at any given height.

#### Whorl information estimation.

**Fig. 2.**
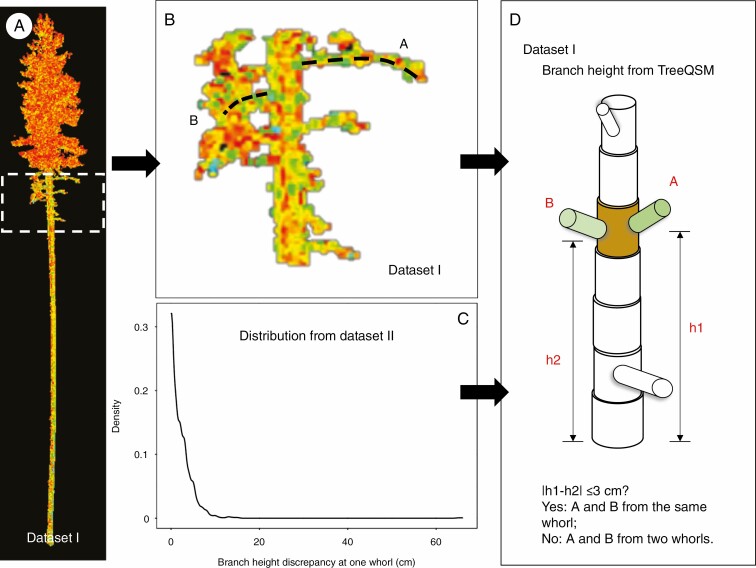
Flowchart detailing the rules of the ‘0’ branch cluster. (A) Cloud points of a scanned sample tree. (B) Details of the branches. (C) By investigating branch information from 4797 whorls (dataset I), the distribution of branch height discrepancy at each whorl was generated. (D) The TreeQSM algorithm can produce cylinders of woody parts that include branch height. Example: from the TreeQSM result, the heights of branches A and B can be derived. We denote them as h1 and h2. If |h1 − h2| ≤ 3 cm, we assume branches A and B are attached to the same whorl. Otherwise they are from two consecutive whorls.

 Based on TreeQSM, the branch architecture was reconstructed by segmenting the point cloud, and the segments provided modelled information for each fitting cylinder. We selected the reconstructed branches that were attached to the trunk (‘0’ branch). The branch height was then determined and linked to the stem taper curve. Given that several branches can be attached to one whorl, we needed to decide whether the ‘0’ branches belonged to the same whorl. By analysing 12 952 branches from 3802 whorls with at least two branches (out of 4797 whorls in total) in 122 Scots pine trees (dataset II), we found that the branches of each whorl were located mostly within 3 cm of each other (Fig. 2C). Hence, TSM_tls_ sets the rule thus: if the ‘0’ branch height of two consecutive branches was <3 cm, then these two branches were clustered in one whorl.

Additionally, from TreeQSM branch extraction results, we found that in the upper crown of sample trees very few or even no branches >1 m were detected, which was not realistic. Previous research has reported that the accuracy of branch extraction is highly dependent on the location of the living branch inside the crown (Pyörälä *et al.*, 2018*a*). Thus, for the upper crown layer, whenever the distance from the topmost whorl to the treetop was >0.5 m, fake whorls were inserted. We optimized the interval of consecutive fake whorls by testing a range of values: 10–30 cm with 5-cm intervals. Using each candidate interval, fake whorls were inserted and branch number at each whorl was estimated (for details of the method see the *Branch number and individual basal area estimation* section). By comparing the branch number with destructive measurements, a 25-cm interval was accepted in the end. Then, whorl height was calculated as the average height of the ‘0’ branches in each whorl.

#### Branch number and individual basal area estimation.

Using the height of each whorl as input, the stem cross-sectional diameter at each whorl was determined using the corresponding stem taper curve. Branch number and the basal area of each individual branch were then estimated using tree structure models (Models 1–3).

#### Model 1.

According to the branch number estimate model proposed by Mäkelä and Mäkinen (2003), the branch number in whorl *i*, $N_{bi}$, was calculated as:


$$\begin{array}{l} N_{bi}=b_{0}+b_{1}ln\left(\Delta h_{i}\right)+\frac{b_{2}\left(H-1.3\right)}{D_{BH}}+b_{3}h_{r}+\varepsilon_{k} \end{array}$$ (1)

where *b*_0_, *b*_1_ and *b*_2_ are parameters, Δ*h*_*i*_ (cm) is the height increment between whorl *i* and whorl *i* − 1, *H* (m) is tree height, *D*_BH_ (cm) is diameter at breast height, *h*_*r*_ is branch relative height in the crown and *ε*_*k*_ is the random error from tree *k*.

#### Model 2.

Based on PMT, as presented by Makela (2002), stem cross-sectional area *A*_*i*_ at the height of whorl *i* was proportional to the sum of branch basal area at and above whorl *i* (from whorl *i* to the first whorl at the top), *B*_*i*_ + *B*_*i*−1_ + … + *B*_1_:


$$\begin{array}{l} B_{i}+B_{i-1}+\ldots +B_{1}=\eta A_{i} \end{array}$$ (2)

where *η* is an empirical coefficient and *B*_*i*_ is the sum of branch basal area at whorl *i*, calculated as follows:


$$\begin{array}{l} B_{i}=\;\;\;(A_{i}-A_{i-1}) \end{array}$$ (3)

#### Model 3.

Since we have the *B*_*i*_ and *N*_*bi*_ estimates, individual branch basal area distribution can be calculated using Model 3, taken from Mäkinen and Mäkelä (2003). Firstly, the relative sizes of the largest (*R*_*i max*_) and smallest (*R*_*i min*_) living branches in whorl *i* were defined as Model 3: the largest and smallest branch basal area in whorl *i* divided by the mean branch basal area in whorl *i*. The models for *R*_*i max*_ and *R*_*i min*_ were estimated using the following equations:


$$\begin{array}{ll} lnR_{i\;max}=\; & b_{0}+b_{1}N_{bi}+b_{2}\left(1.1-h_{ri}\right)\\ & +b_{3}\ln\left(1.1-h_{ri}\right)+b_{4}D_{BH}+b_{5}H/D_{BH}+\varepsilon_{k} \end{array}$$ (4)

$$\begin{array}{ll} \ln\left(\frac{R_{i\;min}}{1-R_{i\;min}}\right)=\; & b_{0}+b_{1}N_{bi}+b_{2}\left(1.1-h_{ri}\right)\\ & +b_{3}\ln\left(1.1-h_{ri}\right)+b_{4}D_{BH}+\varepsilon_{k} \end{array}$$ (5)

where *b*_0–5_ are parameters, *R*_*i*_ is the relative size of the branch in whorl *i*, *N*_*bi*_ is the number of branches in whorl *i*, *h*_*ri*_ is the branch relative height in the crown (distance from the tree top to whorl *i* divided by crown length) and *ε*_*k*_ is the random error from tree *k*. Then, individual branch size was drawn from a uniform distribution and *R*_*i max*_ and *R*_*i min*_ were used as the constraints. For the whorls that were estimated as having only one branch, branch basal area was assumed to be *B*_*i*_. Thus, individual branch diameter can be derived according to branch basal area.

#### Model 4.

 Finally, individual branch biomass was estimated using a mixed linear model as in Lehtonen *et al*. (2004b) and the whole-tree branch wood biomass was the sum of individual branch biomass. Let *w*_*bki*_ be the dry weight of branch i on tree k, then *w*_*bki*_ can be modelled as a function of branch diameter (*d*_*ki*_):


$$\begin{array}{l} \ln w_{bki}\left(d\right)=b_{0}+b_{1}{\left[\ln(d_{ki})\right]}^{0.22}+ln\varepsilon_{k0}+\varepsilon_{k1}{\left[\ln(d_{ki})\right]}^{0.22}+lne_{ki} \end{array}$$ (6)

where *b*_0_ and *b*_1_ are fixed parameters, *ε*_*k*0_ and *ε*_*k*1_ are random tree parameters and *e*_*ki*_ is residual. The total branch wood biomass of each sample tree was determined by summing the biomasses of individual living branches.

### Parameter value estimation

Models 1–4 have been previously tested for Scots pine in south Finland (Mäkelä, 2002; Mäkinen and Mäkelä, 2003; Lehtonen *et al.*, 2004*b*; Hu *et al.*, 2020). The parameter values of Models 1–3 applied in this study were estimated based on dataset II. The parameter values for eqn (6) (Model 4) have been estimated previously using the same VAPU dataset (Lehtonen *et al.*, 2004*b*). For mixed effect models [eqns (1), (4)–(6)], parameters were estimated using the lmer function in R from the lme4 package (Bates *et al.*, 2014) and pseudo-*R*^2^ was calculated using the r.squaredGLMM function in R from the MuMIn package as an evaluation indicator (Nakagawa and Schielzeth, 2013).

### Model evaluation

We used sample trees from dataset I to compare the accuracy of whorl and branch estimates from the TSM_tls_ method (against reference measurements) with the accuracy obtained directly from a TreeQSM algorithm (against reference measurements). The comparison combined: (1) a tree-level variable, i.e. the number of whorls; and (2) whorl-level variables, which included branch number, diameter and biomass (Table 2). To evaluate tree- and whorl-level results, we manually paired individual whorls from the TSM_tls_ method and destructive measurement. To locate each whorl, we paired each measured whorl with the modelled one that was the closest to the measurement with respect to stem cross-sectional area. Since we inserted the fake whorls in the top of the crown, we examined the performance of the TSM_tls_ method separately at the upper, middle and lower crown. We classified the living branches inside the crown into three layers on the basis of relative height in the crown: (1) upper crown <30 %; (2) middle crown 30–70 %; and (3) lower crown 70–100 %. The accuracy, commission error (*E*_c_) and omission error (*E*_o_) of whorl/branch number were defined for each crown layer as well as for the whole tree using the following equations:

**Table 2.** Evaluated tree- and whorl-level information from different approaches

Estimate	Approach	Description	Indicators
*N*_*w*_	TSM_tls_	Whorl setting rules (cluster branch and fake whorls)	Accuracy, *E*_*c*_, *E*_*o*_$E_{o}$
	TreeQSM	Each branch represented one whorl	
*d*_*b*_ (within paired whorls)	TSM_tls_	Model 1 and Model 2	Accuracy, *E*_*c*_, *E*_*o*_$E_{o}$
	TreeQSM	Branch number from TreeQSM result	
*d*_*b*_	TSM_tls_	Model 3	Relative error, RMSE
	TreeQSM	Branch diameter from TreeQSM result	
*w*_*b*_	TSM_tls_	Model 4	KS test
	TreeQSM	Volume from TreeQSM results multiplied by branch wood density	

*N*_*w*_, whorl number; *N*_*b*_, branch number; *d*_*b*_, individual branch diameter; *w*_*b*_, individual branch biomass.


$$\begin{array}{l} Accuracy\;(\;\%\;)=\frac{N_{p}}{N_{p}+N_{c}+N_{o}}\times 100 \end{array}$$ (7)

$$\begin{array}{l} E_{c}\;(\;\%\;)=\frac{N_{c}}{N_{p}+N_{c}+N_{o}}\times 100 \end{array}$$ (8)

$$\begin{array}{l} E_{o}(\;\%\;)=\frac{N_{o}}{N_{p}+N_{c}+N_{o}}\times 100 \end{array}$$ (9)

where *N*_*p*_ is the number of paired whorls/branches, *N*_*c*_ is the number of commission errors, i.e. the number of whorls/branches that were falsely detected or modelled, and *N*_*o*_ is the number of omission errors, i.e. the number of whorls/branches omitted from the measurements. Moreover, the correspondence between measured and estimated individual branch diameter was evaluated by comparing the whorl mean, whorl maximum and whorl minimum diameters with destructive measurements. The two-sample Kolmogorov–Smirnov (KS) test and comparisons of the cumulative distribution function were used to analyse whether the individual branch biomass distributions in three crown layers determined by TreeQSM and TSM_tls_ were different from our destructive measurements.

### Total branch biomass from other TLS-based models

Additionally, we tested the performance of our total branch biomass estimates against tree-level allometric models evaluated with TLS-based input variables. Here we used empirical allometric (denoted QSM_Allometry) (Repola, 2009) and pipe-model based equations (denoted QSM_CROBAS) (Valentine and Mäkelä, 2005; Hu *et al.*, 2020). These two methods utilized the tree height and diameter derived from TreeQSM as inputs with parameter values as presented in previous literature (Table 3). We used linear regression to compare the different approaches with reference to destructive measurements. As general indicators of the accuracy of the model approaches, *R*^2^, root mean square error (RMSE) [eqn (10)] and the coefficient of variation (CV) of the RMSE [eqn (11)] of total branch biomass estimation were calculated. In addition, we also used the concordance correlation coefficient (CCC) (Lawrence and Lin, 1989) to compare agreement of model estimates with reference.

**Table 3.** Model description for the TLS-derived branch woody biomass estimations, including stump diameter (*d*_*S*_, m), wood density (*ρ*_*b*_, kg m^−3^), height (*H*, m), stem cross-sectional area at crown base (*A*_*c*_, m^2^), and parameters used in each equation. QSM_Allometry was based on Repola (2009) and QSM_CROBAS on Hu *et al*. (2020)

Models	Equations	*ρ*_*b*_	*a*	*b*	*c*	*φ*_b_	*η*_*s*_/*η*_*b*_
TreeQSM	$W_{b}=V_{cylinder}\times \rho_{b}$	400	–	–	–	–	–
*QSM_Allometry*	$In(W_{b})=a+b\times \frac{d_{s}}{d_{s}+6}+c\times \frac{H}{H+1}$	–	–6.16	15.08	–2.62	–	–
*QSM_CROBAS*	$W_{b}=\rho_{b}\times \varphi_{b}\times \eta_{s}/\eta_{b}\times A_{c}$	400	–	–	–	1.16	1.65
TSM_tls_*	$W_{b}=\sum_{1}^{i}W_{bj}$	–	–	–	–	–	–

*Total branch biomass from TSM_tls_ was calculated by summing individual branch biomass (*w*_*bj*_, kg) based on Model 4, eqn (6).


$$\begin{array}{l} RMSE\;(kg)=\sqrt{\frac{\overset{n}{\underset{1}{\sum }}{\left({W_{b}}_{model}-{W_{b}}_{ref}\right)}^{2}}{n}} \end{array}$$ (10)

$$\begin{array}{l} CV\;RMSE\;(\;\%\;)=\frac{RMSE}{\overset{n}{\underset{1}{\sum }}{W_{b}}_{ref}/n}\times 100 \end{array}$$ (11)

## RESULTS

### Model parameter values

In this study, parameters of Models 1–4 were estimated based on dataset II (Table 4). For linear mixed models [eqns (1), (4)–(6)], only the fixed part was applied to the TSM_tls_ method for estimating branch number, diameter and biomass.

**Table 4.** Parameter estimate and CV of each parameter in Models 1–4. Numbers in parentheses were the modelling sample numbers from dataset II used to estimate the parameters

Parameter	Eqn (1) (4797)		Eqns (2) and (3) (4797)		Eqn (4) (4797)		Eqn (5) (4797)		Eqn (6) (13 984)* ^1^	
	Estimate	CV	Estimate	CV	Estimate	CV	Estimate	CV	Estimate	CV
Fixed part										
η	–		1.810	0.002	–		–		–	
$b_{0}$	1.514	0.197	–	–	0.237	0.249	0.894	0.210	–36.100	0.011
$b_{1}$	0.883	0.041	–	–	0.079	0.063	–0.412	0.058	32.514	0.010
$b_{2}$	–0.712	0.567	–	–	–0.277	0.097	1.404	0.106	–	–
$b_{3}$	–0.405	0.163	–	–	0.036	0.194	–0.214	0.187	–	–
$b_{4}$	–	–	–	–	0.001	0.000	–0.004	0.000	–	–
$b_{5}$	–	–	–	–	–0.007	0.571	–	–	–	–
Random part										
$\varepsilon_{k}{\left(\varepsilon_{k0}\right)}^{*2}$	0.578	0.019	–	–	0.005	0.254	0.060	0.072	0.048	0.002
$\varepsilon_{k1}$	–	–	–	–	–	–	–	–	5.846	0.005
Residual	1.109	0.014	–	–	0.043	0.086	0.989	0.018	0.182	0.001
Whole-model evaluation										
$R_{m}^{2}$* ^3^	0.246	–	0.980	–	0.457	–	0.223	–	0.890	–
$R_{c}^{2}$	0.421	–	–	–	0.554	–	0.256	–	0.910	–
RMSE	1.097	–	0.003	–	0.219	–	1.023	–	1.814	–

* ^1^Parameters were estimated by Lehtonen *et al.* (2004*b*) using the same dataset; ^*2^*ε*_k0_ and *ε*_k1_ were only applied to eqn (6); * ^3^$R_{m}^{2}$ is the marginal *r*^2^ value representing the variance explained by the fixed effects; $R_{c}^{2}$ is the conditional *r*^2^ value representing the variance explained by the entire model, including both fixed and random effects.

### Whorl number evaluation

**Fig. 3.**
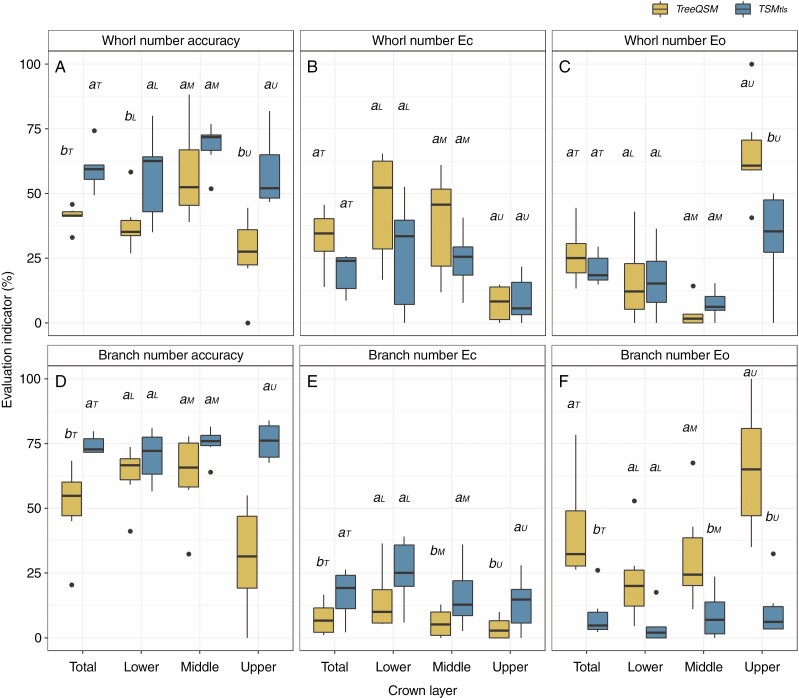
Box plots showing accuracy, commission error (*E*_*c*_) and omission error (*E*_*o*_) for whorl (A, B, C) and branch number (D, E, F) estimates in the whole tree and in different crown layers using TreeQSM and TSM_tls_ approaches. Box dimensions show the quartiles for 25–75 % accuracy, the black line represents median indicator values and the whiskers show minimum and maximum values. Lower-case letters a, b on the boxplots show the significance of each indicator between different approaches (*P* < 0.05) and subscripts T, L, M and U represent the whole tree and different crown layers.

Whorl number was estimated for sample trees in dataset I using three approaches (Table 2). The TSM_tls_ method improved whorl number estimate accuracy from 40.43 to 57.73 % compared with the TreeQSM method, and the upper crown showed the highest improvement of accuracy, from 29.20 to 52.59 % (Table 5, Fig. 3). In addition, commission error (*E*_*c*_) and omission error (*E*_*o*_) were reduced by 14.38 and 3.01 percentage points, respectively (Fig. 3A–C, Table 5).

**Table 5.** Paired whorl number (*N*_*p*_), commission number (*N*_*c*_) and omission number (*N*_*o*_) between estimated whorl number and reference destructive measurement at different crown layers and whorl tree levels, with TEM_tls_ and TreeQSM methods. Whorl number accuracy, commission error (*E*_*c*_) and omission error (*E*_*o*_) are also listed

Tree no.	Crown layer	Ref.	TreeQSM							TSM_tls_						
			Total	*N*_*p*_	*N*_*c*_	*N*_*o*_	Accuracy (%)	*E*_*c*_ (%)	*E*_*o*_ (%)	Total	*N*_*p*_	*N*_*c*_	*N*_*o*_	Accuracy (%)	*E*_*c*_ (%)	*E*_*o*_ (%)
11	Total	32	20	15	5	17	40.54	13.89	44.44	29	26	3	6	74.29	8.57	17.14
	Upper	9	0	0	0	9	0.00	0.00	100.00	11	9	2	0	81.82	18.18	0.00
	Middle	12	12	10	2	2	71.43	14.29	14.29	11	10	1	2	76.92	7.69	15.38
	Lower	11	8	5	3	6	35.71	21.43	42.86	7	7	0	4	63.64	0.00	36.36
12	Total	41	55	29	26	12	43.28	38.81	17.91	46	32	14	9	58.18	25.45	16.36
	Upper	13	5	5	0	8	38.46	0.00	61.54	9	9	0	4	69.23	0.00	30.77
	Middle	16	30	16	14	0	53.33	46.67	0.00	22	16	6	0	72.73	27.27	0.00
	Lower	12	20	8	12	4	33.33	50.00	16.67	15	7	8	5	35.00	40.00	25.00
31	Total	49	78	37	41	12	41.11	45.56	13.33	53	36	17	13	54.55	25.76	19.70
	Upper	23	16	12	4	11	44.44	14.81	40.74	15	13	2	10	52.00	8.00	40.00
	Middle	16	41	16	25	0	39.02	60.98	0.00	25	14	11	2	51.85	40.74	7.41
	Lower	10	21	9	12	1	40.91	54.55	4.55	13	9	4	1	64.29	28.57	7.14
32	Total	41	45	27	18	14	45.76	30.51	23.73	46	33	13	8	61.11	24.07	14.81
	Upper	18	5	4	1	14	21.05	5.26	73.68	17	12	5	6	52.17	21.74	26.09
	Middle	15	17	15	2	0	88.24	11.76	0.00	16	13	3	2	72.22	16.67	11.11
	Lower	8	23	8	15	0	34.78	65.22	0.00	13	8	5	0	61.54	38.46	0.00
41	Total	55	51	31	20	24	41.33	26.67	32.00	43	37	6	18	60.66	9.84	29.51
	Upper	29	14	9	5	20	26.47	14.71	58.82	15	14	1	15	46.67	3.33	50.00
	Middle	16	28	15	13	1	51.72	44.83	3.45	20	15	5	1	71.43	23.81	4.76
	Lower	10	9	7	2	3	58.33	16.67	25.00	8	8	0	2	80.00	0.00	20.00
42	Total	54	67	30	37	24	32.97	40.66	26.37	52	34	18	20	47.22	25.00	27.78
	Upper	31	14	10	4	21	28.57	11.43	60.00	16	14	2	17	42.42	6.06	51.52
	Middle	14	29	13	16	1	43.33	53.33	3.33	19	13	6	1	65.00	30.00	5.00
	Lower	9	24	7	17	2	26.92	65.38	7.69	17	7	10	2	36.84	52.63	10.53
Total	All	272	316	169	147	103	40.33	35.17	24.40	269	198	71	74	57.73	20.70	21.57

### Branch information evaluation

#### Branch number (Model 1).

 With the TSM_tls_ method, branch number was estimated for each individual whorl. For the 199 paired whorls, the TSM_tls_ method had an overall accuracy of 68.60 % and registered its highest improvement (from 28.94 to 68.92 %) compared with the TreeQSM method in the upper layer of the crown (Table 6). Using the TSM_tls_ method, *E*_*c*_ and *E*_*o*_ relative to the destructively measured branches were 22.24 and 9.16 %, respectively. The inserted fake whorls in the upper crown led to more commission errors (42 more cases) than TreeQSM, but the respective omission error decreased almost 6-fold (97 fewer cases) (Table 6).

**Table 6.** Paired branch number (*N*_*p*_), commission number (*N*_*c*_) and omission number (*N*_*o*_) between estimated whorl number and reference destructive measurement at different crown layers and whorl tree levels, with TSM_tls_ and TreeQSM approaches. Whorl number accuracy, commission error (*E*_*c*_) and omission error (*E*_*o*_) are also listed

Tree no.	Crown Layer	Ref.	TreeQSM							TSM_tls_						
			Total	*N*_*p*_	*N*_*c*_	*N*_*o*_	Accuracy (%)	*E*_*c*_ (%)	*E*_o_ (%)	Total	*N*_*p*_	*N*_*c*_	*N*_*o*_	Accuracy (%)	*E*_*c*_ (%)	*E*_*o*_ (%)
11	Total	87	19	18	1	69	20.45	1.14	78.41	69	65	4	22	71.43	4.40	24.18
	Up	37	0	0	0	37	0.00	0.00	100.00	28	28	0	9	75.68	0.00	24.32
	Middle	34	11	11	0	23	32.35	0.00	67.65	27	25	2	9	69.44	5.56	25.00
	Lower	16	8	7	1	9	41.18	5.88	52.94	14	12	2	4	66.67	11.11	22.22
12	Total	81	38	37	1	44	45.12	1.22	53.66	86	69	17	12	70.41	17.35	12.24
	Up	29	5	5	0	24	17.24	0.00	82.76	28	24	4	5	72.73	12.12	15.15
	Middle	35	20	20	0	15	57.14	0.00	42.86	37	30	7	5	71.43	16.67	11.90
	Lower	17	13	12	1	5	66.67	5.56	27.78	21	15	6	2	65.22	26.09	8.70
31	Total	70	59	49	10	21	61.25	12.50	26.25	79	61	18	7	70.93	20.93	8.14
	Up	18	13	11	2	7	55.00	10.00	35.00	24	18	6	0	75.00	25.00	0.00
	Middle	34	29	24	5	10	61.54	12.82	25.64	40	31	9	1	75.61	21.95	2.44
	Lower	18	17	14	3	4	66.67	14.29	19.05	15	12	3	6	57.14	14.29	28.57
32	Total	50	42	32	10	18	53.33	16.67	30.00	74	48	26	2	63.16	34.21	2.63
	Up	20	5	5	0	15	25.00	0.00	75.00	26	18	8	2	64.29	28.57	7.14
	Middle	16	16	14	2	2	77.78	11.11	11.11	25	16	9	0	64.00	36.00	0.00
	Lower	14	21	13	8	1	59.09	36.36	4.55	23	14	9	0	60.87	39.13	0.00
41	Total	63	45	39	6	24	56.52	8.70	34.78	91	63	28	0	69.23	30.77	0.00
	Up	27	13	11	2	16	37.93	6.90	55.17	41	27	14	0	65.85	34.15	0.00
	Middle	28	23	21	2	7	70.00	6.67	23.33	35	28	7	0	80.00	20.00	0.00
	Lower	8	9	7	2	1	70.00	20.00	10.00	15	8	7	0	53.33	46.67	0.00
42	Total	67	46	43	3	23	62.32	4.35	33.33	87	61	26	6	65.59	27.96	6.45
	Up	25	11	10	1	15	38.46	3.85	57.69	39	24	15	1	60.00	37.50	2.50
	Middle	28	23	22	1	5	78.57	3.57	17.86	32	26	6	2	76.47	17.65	5.88
	Lower	14	12	11	1	3	73.33	6.67	20.00	16	11	5	3	57.89	26.32	15.79
Total	All	418	249	218	31	199	48.66	6.92	44.42	486	367	119	49	68.60	22.24	9.16

#### Branch diameter and biomass (Models 2, 3 and 4).

**Fig. 4.**
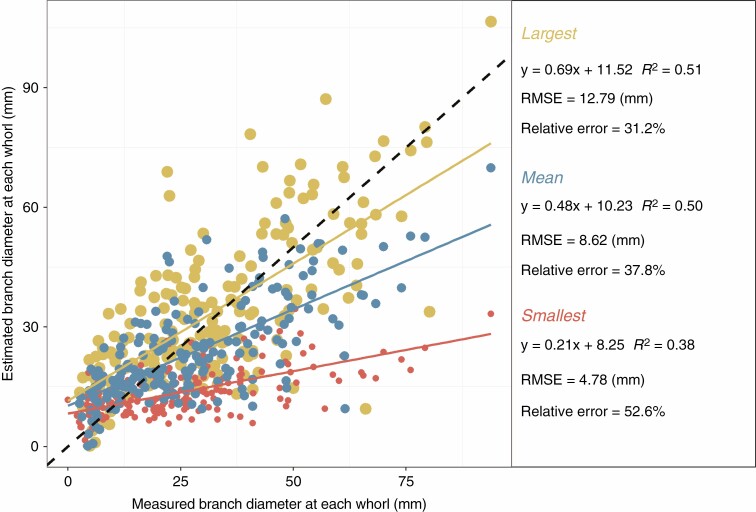
Comparison of destructive measurement- and TSM_tls_-based individual branch diameter (*n* = 594). Large, small and medium dots denote whorl maximum, minimum and mean diameter, respectively.

**Fig. 5.**
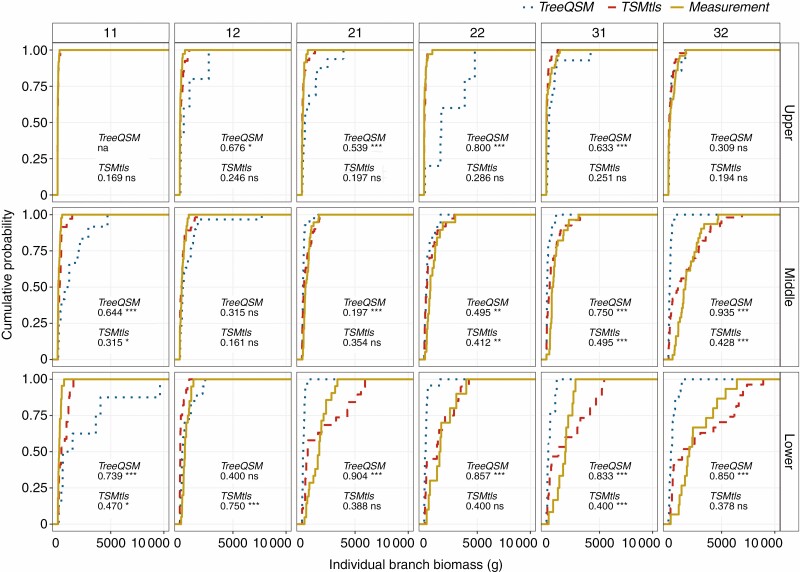
Comparison of the empirical cumulative distribution of individual branch biomass among TreeQSM, TSM_tls_ and measurements presented by different crown layers in the six test trees (tree ID number is in the box at the top of each panel). Values in the figure are the *D* statistic values from the KS test, which quantify the maximum vertical distances between TreeQSM*/*TSM_tls_-based distribution and measurement distribution. Asterisks denotes the significance level in the KS test: ns, not significant; *P < 0.05; **P < 0.01; ***P < 0.001. In the upper crown of tree 11 no branches were extracted from TreeQSM, and therefore the KS test was not applied to TreeQSM (marked as na).

 Within paired whorls, the diameter estimation of the largest branch had a lower relative error (31.2 %) than the smallest branch estimation (52.6 %) (Fig. 4). Nevertheless, both models [eqns (5) and (6)] underestimated the branch diameter, and the underestimation was more pronounced on the smallest individual in each whorl, with a proportion of 73.8 % of the smallest branches being underestimated. Furthermore, the empirical cumulative distribution function (ECDF) of individual branch biomass from the TSM_tls_ method showed that the bias occurred more in the middle and lower part of the crown (Fig. 5, *P* < 0.05).

**Fig. 6.**
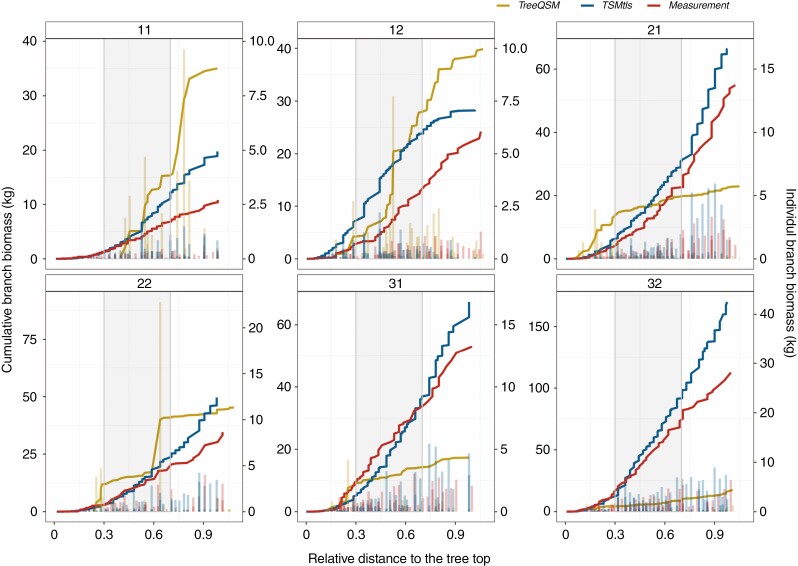
Comparison of cumulative (lines, primary *y*-axis) and individual branch (histograms, secondary *y*-axis) biomass estimates from TreeQSM, TSM_tls_ and destructive measurements at relative height of each sample tree (tree ID number is in the box at the top of each panel). The grey area shows the middle part of the crown.

Although branch diameters were underestimated, the cumulative branch biomass estimated by the TSM_tls_ method agreed much better with the destructive measurements than the TreeQSM method (Fig. 6, Table 7) at the tree level. Moreover, the KS test showed that individual branch estimates produced by the TSM_tls_ method had lower *D* statistic values than the TreeQSM method when compared with destructive measurements in all the crown layers of each sample (Fig. 5). It indicated a significant improvement of the individual branch biomass estimate using the TSM_tls_ method. For the TreeQSM method, some extreme individual branch biomass estimates (Fig. 6) and unobserved branches (Fig. 3C, tree 11) could be the main reasons behind the bias of the total branch biomass (Table 7).

**Table 7.** Summary of total branch biomass estimates from TLS-based models. For *R*^2^, RMSE, CV RMSE and CCC, *n* = 6. Slope and intercept values come from the regression models between TLS-based models and measurements. Models are arranged according to performance from the worst to the best

Model	Slope	Intercept	*R* ^2^	RMSE (kg)	CV RMSE ( %)	CCC
TreeQSM	−2.46	112.2	0.27	30.37	57.45	0.43
QSM_Allometry	2.26	−11.90	0.91	10.58	20.02	0.95
QSM_CROBAS	0.69	1.79	0.96	6.86	12.97	0.98
TSM_tls_	0.72	5.08	0.98	5.10	9.66	0.99

#### Total branch biomass from TLS-based models.

 When compared with destructive branch biomass measurements, the TSM_tls_ method showed 1.76 and 25.27 kg lower RMSE and 3.31 and 47.79 percentage points lower CV RMSE than the QSM_CROBAS and TreeQSM methods. The performance of the QSM_Allometry method was intermediate between the QSM_CROBAS and TreeQSM methods. The TSM_tls_ method was closer to the 1:1 line and the agreement between TSM_tls_ estimates and destructive measurements expressed as CCC was higher (0.99) than those of the other TLS-based models (0.43–0.98; Table 7).

## DISCUSSION

This study proposes a method to estimate branch biomass by linking TLS data with tree structure models. The results showed that the TSM_tls_ method of estimating total branch biomass is more accurate than the other TLS-based approaches tested.

### Individual branch extraction from TLS

The TreeQSM method has been widely used in tree structure or biomass estimation (Gonzalez De Tanago *et al.*, 2017; Lau *et al.*, 2018, 2019). Our main rationale for developing the method further was its inaccurate individual branch extraction. Even though several scans were taken from different points around the target trees to gain more comprehensive branch information, many of the branches were still not observed, especially in the upper part of the crown, which showed higher omission error than the lower part (Figs 3C and 6). This has been observed in previous studies as well (Eysn *et al.*, 2013; Boudon *et al.*, 2014) and it was mainly caused by the scanner’s features (time-of-flight scanner): the signal will return as soon as it reaches a non-penetrable obstacle, rendering the cloud points unavailable for locations not visible from the scanner. As the lower branches may hinder visibility, fewer signals will reach the upper branches, which may cause branch omission. Moreover, the upper canopy is further from the scanner, which makes the point cloud naturally sparser compared with the lower parts.

In addition, in TreeQSM results it is not hard to notice the abnormal outliers that feature extremely large individual branch biomass (Fig. 6). This is because the main task of TreeQSM is to reconstruct the woody part as cylinders (Raumonen *et al.*, 2013). To get an accurate result, this procedure needs enough points, and fewer points increase the likelihood of misevaluated branch diameters. Weather effects such as wind can result in noisy data while scanning and detract from the ability to reconstruct individual branch structures. Additionally, our sample trees are Scots pine, which have needles all year round, and this makes it harder to get the leaf-off scanned data.

### TSM_tls_ method considerations

The TSM_tls_ method relies on the PMT-based equation, in which the stem cross-sectional basal area and branch basal area at each whorl are linearly related [eqn (2)], which has been demonstrated in previous studies (Makela, 2002; Kantola and Mäkelä, 2004, 2006). This linear relationship was used in Model 2, and, together with Model 1, individual branches were simulated in each whorl. It is not surprising that the total branch biomass estimates were in closer agreement with destructive measurements using TSM_tls_ than the TreeQSM method (Table 7), because many branches failed to be observed when using the TreeQSM method (Fig. 3). Also, in the TSM_tls_ method we optimized the interval value of consecutive fake whorls based on destructive measurements, which could also contribute to better results compared with the TreeQSM method. This means that an optimized interval value of consecutive whorls may need to be considered properly when TSM_tls_ is applied to another dataset. Nevertheless, the individual branch number and diameter estimates were still biased using TSM_tls_ (Figs 3 and 4). One reason could be that the branch number model we used was developed on the basis of branch data from trees in the age range of 22–76 years (Mäkelä and Mäkinen, 2003), while the age range of the trees sampled (dataset I) was 91–113 years and dataset II comprised trees of various ages in our study. In eqn (1), annual height growth and slenderness were considered the main variables reported to be affected by tree age (Hann and Larsen, 1991; Helmisaari *et al.*, 2002; Weiskittel *et al.*, 2011), and it is possible that the age influenced the accuracy of the model. Additionally, branch growth is also affected by shading or interaction with other branches and trees, especially for the branches in the middle or lower part of the crown. This could result in fewer branches in reality than modelled and is also consistent with our result that the modelled individual branch biomass distribution had more bias in the lower than the upper crown (Fig. 5). Hence, the overestimated branch number leads to a smaller mean branch basal area for a given whorl total, which may be one reason why the branch basal area was underestimated (Fig. 4). Nevertheless, the accuracy of the branch number estimate (68.60 %) was improved compared with previous studies: Pyörälä *et al.* (2018*a*) extracted individual Scots pine branches with an accuracy of 64.8 % and Pyörälä *et al.* (2018*b*) reported a higher branch number detection accuracy (69.9 %), but they only considered the largest branches of whorls, where stem diameter exceeded 15 cm. Other studies focusing on individual branch information also indicate that the larger the branch, the easier it is to detect, even if only relatively large branches are considered (Lau *et al.*, 2018).

The present TSM_tls_ approach has been motivated by the convenience of TLS and the idea of PMT-based carbon allocation (Mäkelä and Mäkinen, 2003; Mäkinen and Mäkelä, 2003). This method allows branch biomass estimation without destructively harvesting the trees. The results are encouraging; however, only a limited dataset was used for validation. In order to apply this method in operational work, a larger dataset would be required to confirm our results in the future.

### Tree-level branch biomass estimation by different methods

At the whole-tree level, four different TLS-based methods to predict the total branch biomass were compared. Despite the notable underestimation of individual branch diameter and biomass in the paired whorls, the total branch biomass estimates obtained using the TSM_tls_ method showed close agreement with the measured trees (Fig. 6) and this method performed better than the other TLS-based methods (Table 7). This is in line with the result that the estimate of total basal area of branches at each whorl (Model 2) was unbiased, as predicted by the PMT (Shinozaki *et al.*, 1964*b*), and suggests that it is more accurate for total biomass estimation than individual branch attributes.

In contrast, traditional allometry models for estimating total branch biomass are based on breast height or stump diameter and tree height (Kärkkäinen, 2005; Repola *et al.*, 2007). Although these models have been applied to different species and sites, and the variables used in the models are easy to measure and extract from TLS data, the reliability and applicability of the estimates still depend on data from different studies (Repola, 2009). Parresol (1999) found that the total branch biomass varies from stand to stand even with the same tree height and trunk diameter due to different growth conditions, reducing the precision of the biomass estimate using allometry models. Though Repola (2008) has developed multivariate mixed models to estimate tree component biomass better by considering simultaneous correlation between different tree components (foliage, branch, stem and root), it is still a challenge to use TLS data alone to obtain biomass estimation models for each tree component. From this perspective, it is not surprising that a PMT-based model together with TLS data could provide a more reliable and accurate branch biomass estimate than allometry models.

### Potential application of TSM_tls_

This study proposes a new method to estimate branch biomass based on TLS data with tree structure models, which provides a non-destructive means of investigating the vertical branch distribution in the crown. Firstly, as the basis of branch attribute estimation, PMT-based tree structure models have been tested for Norway spruce and silver birch in previous studies (Ilomäki *et al.*, 2003; Kantola and Mäkelä, 2004; Kantola and Mäkelä, 2006; Hu *et al.*, 2020). This suggests that we could apply our method to more species, such as Norway spruce, which require greater effort in field measurements due to their dense foliage. Secondly, recent advances in TLS data processing have enabled us to produce relatively accurate and unbiased stem taper curves in boreal forests (Pitkänen *et al.*, 2019, 2021), which further contributes to the estimation of branch basal areas of each whorl. Although our scanned sample trees (dataset I) are limited in number and focused on Scots pine only, TSM_tls_ still has potential and value in measuring more species and over a larger area.

### CONCLUSIONS

In this study we present a new method that not only estimates branch biomass precisely, but also present an opportunity to estimate individual branch attributes using TLS data. The TSM_tls_ method presented showed greater accuracy of tree-level branch biomass estimation than other TLS-based methods. Although a high number of commission errors appeared in whorl/branch number estimates and biases could be found in branch diameter estimates, TSM_tls_ produced more accurate results for whorl-level information estimates than TreeQSM. The good performance in Scots pine trees shows the great potential in extending the method to more species and larger areas. While our results are based on six TLS-scanned trees, a limited dataset compared with other studies, it would be necessary to collect a larger dataset to confirm our conclusions in the future.
